# Automatic Seizure Detection Based on Stockwell Transform and Transformer

**DOI:** 10.3390/s24010077

**Published:** 2023-12-22

**Authors:** Xiangwen Zhong, Guoyang Liu, Xingchen Dong, Chuanyu Li, Haotian Li, Haozhou Cui, Weidong Zhou

**Affiliations:** 1 School of Integrated Circuits, Shandong University, Jinan 260100, China; 202212333@mail.sdu.edu.cn (X.Z.); gyliu@sdu.edu.cn (G.L.); 202132378@mail.sdu.edu.cn (X.D.); 202232355@mail.sdu.edu.cn (C.L.); 202212327@mail.sdu.edu.cn (H.L.); 202232343@mail.sdu.edu.cn (H.C.); 2 Shenzhen Institute, Shandong University, Shenzhen 518057, China

**Keywords:** automatic seizure detection, transformer, stockwell transform, EEG

## Abstract

Epilepsy is a chronic neurological disease associated with abnormal neuronal activity in the brain. Seizure detection algorithms are essential in reducing the workload of medical staff reviewing electroencephalogram (EEG) records. In this work, we propose a novel automatic epileptic EEG detection method based on Stockwell transform and Transformer. First, the S-transform is applied to the original EEG segments, acquiring accurate time-frequency representations. Subsequently, the obtained time-frequency matrices are grouped into different EEG rhythm blocks and compressed as vectors in these EEG sub-bands. After that, these feature vectors are fed into the Transformer network for feature selection and classification. Moreover, a series of post-processing methods were introduced to enhance the efficiency of the system. When evaluating the public CHB-MIT database, the proposed algorithm achieved an accuracy of 96.15%, a sensitivity of 96.11%, a specificity of 96.38%, a precision of 96.33%, and an area under the curve (AUC) of 0.98 in segment-based experiments, along with a sensitivity of 96.57%, a false detection rate of 0.38/h, and a delay of 20.62 s in event-based experiments. These outstanding results demonstrate the feasibility of implementing this seizure detection method in future clinical applications.

## 1. Introduction

Epilepsy, caused by abnormal discharge of brain neurons, affects more than 50 million people worldwide [1]. Epilepsy is characterized by recurrent and sudden seizures, which may cause temporary loss of consciousness or perception, and involuntary body convulsions. Persistent and recurrent seizures can greatly disturb the patients’ life and even endanger their safety. As a fundamental tool for studying the human brain, electroencephalogram (EEG) has become an important tool for assisting the clinical diagnosis of neurological diseases [2,3,4,5]. Presently, epileptic seizure events are mainly annotated by neurology experts based on clinical experience through analyzing long-term EEG recordings, which is time-consuming and laborious. Therefore, the development of automatic seizure detection systems, which can reduce the burden on medical staff and assist in patient treatment, has become a valuable research topic.

The research on automatic seizure detection has a history of several decades, and many promising results and preliminary applications have been achieved. One of the earliest seizure detection systems was proposed by Gotman [6] in the early 1980s. He extracted slope, rhythmicity, and sharpness as classification features from the brainwave signals decomposed into half-waves. Later, Gotman [7] and Qu [8] improved the method by developing a patient-specific false alarm model. Subsequently, many time-domain [9,10,11,12], frequency-domain [13,14,15,16,17,18], and deep learning methods [19,20,21] have been developed for seizure detection. For example, Acharya et al. [22] applied convolutional neural network (CNN) for the identification of epileptic EEG signals. Dong et al. [23] proposed an attention-based graph residual network with a redesigned focal loss function to address the class imbalance issue in epileptic seizure detection tasks. In the study of Tsiouris et al. [24], the LSTM model was used to classify EEG features extracted in time and frequency domains.

As EEG signals are typical non-stationary time-series signals, time-frequency analysis approaches such as Short-Time Fourier Transform, Wavelet Transform, and Empirical Mode Decomposition have been commonly employed to generate time-frequency representations for EEG signals [25,26,27]. Stockwell transform (S-transform), proposed by Stockwell et al. [28], is a combined approach of short-time Fourier transform and wavelet transform, allowing for multi-resolution analysis of time series with relatively low computational complexity. S-transform has been widely applied in various fields such as cardiac sound segmentation [29], power quality analysis [30,31,32], medical imaging [33], etc. Recently, researchers attempted to combine the S-transform with traditional classifiers and deep learning-based models for seizure detection, showing its effectiveness in analyzing epileptic EEG signals [34,35,36]. Therefore, in this study, the S-transform was adopted for accurate time-frequency representation of EEG signals.

The Transformer model with self-attention mechanisms was initially designed for machine translation [37]. Currently, it is widely used not only in natural language processing but also in areas such as computer vision [38], speech recognition [39], and motion imaging [40]. Multi-channel EEG signals are typical time series signals and also can be seen as an image, making them suitable for processing with Transformer models. Sun et al. [41] conducted experiments combining Transformer and 3D convolutional neural networks on three emotional EEG datasets, achieving better emotion recognition accuracy than other methods. Yan et al. [42] presented a model combining short-time Fourier transform and Transformer, demonstrating that their model can effectively utilize the time, frequency, and channel information in EEG signals to improve seizure prediction accuracy. Li et al. [43] introduced a novel graph neural network called the spatial-temporal graph attention network with a Transformer encoder (STGATE) for learning graph representations of emotion EEG signals and improving emotion recognition performance. The above studies indicate that Transformer has potential capabilities in EEG signal classification tasks.

This work proposes an effective method for seizure detection by the combination of S-transform and Transformer. Compared with short-time Fourier transform (STFT) and wavelet transform (WT), S-transform has the advantage of STFT and WT while maintaining lower computational complexity. In this work, the time-frequency matrices obtained by S-transform are compressed in specific frequency bands and then inputted into Transformer for automatic feature selection and classification. Transformer-based methods improve performance by assigning different weights to each channel of EEG signals while also increasing the interpretability of the model. The proposed Transformer contributes to improve performance by assigning different weights to each EEG channel while also increasing the interpretability of the model. The performance of the proposed approach is evaluated on the CHB-MIT epileptic EEG database. To the best of our knowledge, this is the first attempt in which S-transform and Transformer have been combined for seizure detection. Experimental results demonstrate the effectiveness of the proposed algorithm.

The rest of the article is organized as follows. Section 2 introduces the method for epileptic seizure detection, which includes S-transform, Transformer, and post-processing. Section 3 describes the CHB-MIT scalp epileptic EEG dataset and experimental results based on segment-level and event-level. Section 4 is devoted to discussing the results and comparing the performance with other algorithms. Finally, Section 5 presents the conclusion.

## 2. Methods

**Figure 1:**
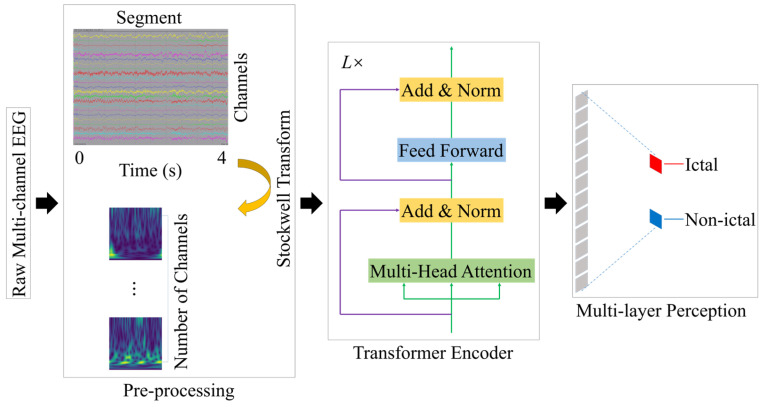
The workflow of the proposed method for seizure detection.

Figure 1 shows the overall workflow of the proposed seizure detection method, which mainly consists of three essential parts: pre-processing (segmentation and S-transform), Transformer, and multi-layer perception. In this work, the multi-channel EEG recordings were divided into 4-s (1024-point) segments.

### 2.1. Stockwell Transform

S-transform is a time-frequency domain analysis method proposed by geophysicist Stockwell [28] in 1996. By combining the advantages of short-time Fourier transform (STFT) and wavelet transform (WT), it has become an effective tool for analyzing and processing non-stationary EEG signals. The S-transform spectrogram $S_{x}(\tau ,\;f)$ of time domain signal x(t) is defined by:

(1) $$S_{x}\left(\tau ,\;f\right)=e^{-i2\pi f\tau }W_{x}(\tau ,\;d)$$

(2) $$W_{x}\left(\tau ,\;d\right)=\int_{-\infty }^{+\infty }x(t)\omega (t-\tau ,\;d)\mathrm{dt}$$

where $W_{x}(\tau ,\;d)$ denotes the wavelet transform of x(t) and ω(t, f) is the mother wavelet, which is defined as:

(3) $$\omega \left(t,\;f\right)=\frac{\left|f\right|}{\sqrt{2\pi }}e^{\frac{-t^{2}f^{2}}{2}}e^{-i2\pi \mathrm{ft}}$$

**Figure 2:**
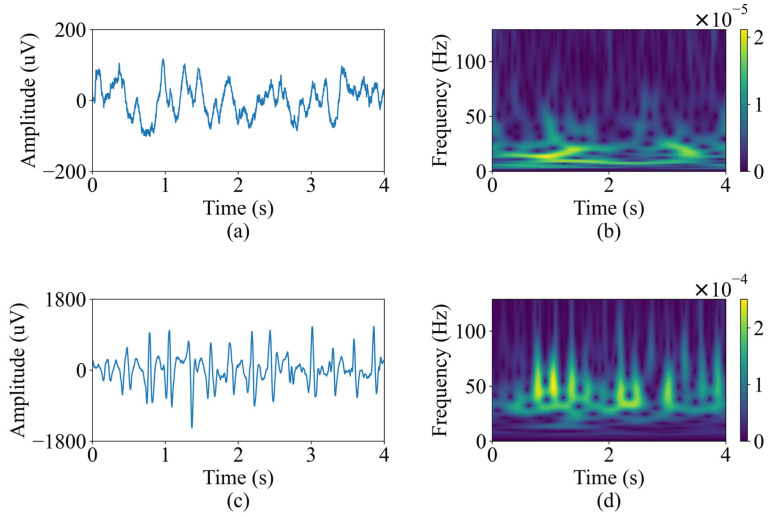
Non-seizure and seizure EEG signals and their S-transform spectrograms. (**a**) Non-ictal EEG signal. (**b**) S-transform of non-ictal EEG. (**c**) Ictal EEG signal. (**d**) S-transform of ictal EEG.

Ultimately, the S-transform can be given as follows:

(4) $$S_{x}\left(\tau ,f\right)=\int_{-\infty }^{+\infty }x(t)\frac{\left|f\right|}{\sqrt{2\pi }}e^{\frac{{-(\tau -t)}^{2}f^{2}}{2}}e^{-i2\pi \mathrm{ft}}\mathrm{dt}$$

where the x(t) represents the segmented 4-s EEG signals in this study. Each EEG segment processed by S-transform returns a time-frequency matrix with size of 128 × 1024, where 128 represents the frequency range from 1 to 128 Hz, and 1024 expresses the time points. Figure 2 shows a 4-s segment of typical non-ictal EEG and a 4 s segment of typical epileptic EEG selected from patient 5, along with the corresponding S-transform spectrograms. It is evident from the figure that not only is the amplitude of the epileptic EEG significantly higher than the non-ictal EEG, but there is also a notable difference in energy between the two signals in the frequency range of 20 to 50 Hz.

**Figure 3:**
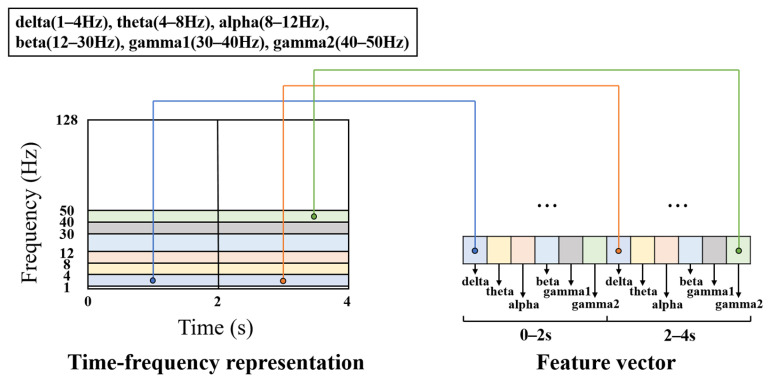
Schematic diagram for compression of EEG time-frequency representation obtained with S-transform in a single channel. Different colors represent corresponding EEG rhythms, including delta (1–4 Hz), theta (4–8 Hz), alpha (8–12 Hz), beta (12–30 Hz), gamma1 (30–40 Hz) and gamma2 (40–50 Hz).

Considering that epileptic EEG signals are concentrated in the frequency range of 3 to 30 Hz [35], the frequency range of 1–50 Hz is selected in order to eliminate power frequency interference, and then divided into 6 sub-bands: delta (1–4 Hz), theta (4–8 Hz), alpha (8–12 Hz), beta (12–30 Hz), gamma1 (30–40 Hz), and gamma2 (40–50 Hz). For a 4-s EEG segment, the 4-s time axis is partitioned into two parts with a 2-s interval. Hence, the time-frequency matrix obtained with S-transform within 1–50 Hz is divided into 12 sub units. The summation of the squared moduli of S-transform in each unit are sequentially concatenated to obtain a feature map of size n × 12 as input to the model, where *n* represents the number of channels. Figure 3 depicts the above time-frequency compression process for a single channel.

### 2.2. Transformer

The Transformer network was originally designed for machine translation, and the sequences fed into the network need to be pre-embedded to obtain a matrix of shape number of words × embedding dimension. We regard each channel of the EEG recording as a word in a sequence. Therefore, the above process is also known as “channel embedding” [44]. In this study, since we want to keep the channel order of each EEG recording consistent, no position encoding was performed on channels.

**Figure 4:**
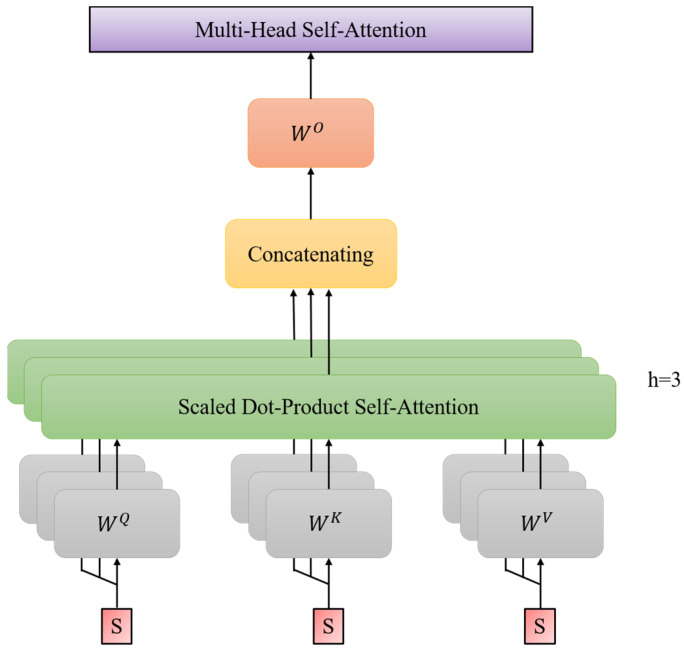
Structure diagram of multi-head self-attention mechanism.

Given that the goal of epilepsy detection task was actually a classification task, we only used the encoder module of the Transformer, which was stacked *L* times. The S-transformed and compressed EEG signal *S* serves as the input feature map of the Transformer encoder. The Transformer encoder consists of two parts: Multi-head Self-Attention (MSA) and Multi-Layer Perception (MLP). Both parts use layer normalization, and their outputs adopt residual connection structures. The self-attention mechanism can be described as a mapping from a query matrix (*Q*) to a set of key (*K*)-value (*V*) pairs. *Q* and *K* have a dimension of $d_{k}$, and *V* has a dimension of $d_{v}$. The output of the sequence *S* after the self-attention mechanism can be calculated by the following equation:

(5) $$\left[\begin{array}{ccc} Q, & K, & V \end{array}\right]=S\left[\begin{array}{ccc} W^{Q}, & W^{K}, & W^{V} \end{array}\right]$$

(6) $$\mathrm{SA}(S)=\mathrm{softmax}({\mathrm{QK}}^{T}/\sqrt{d_{k}})V$$

where $W^{Q}\in R^{d_{\mathrm{model}}\times d_{k}}$, $W^{K}\in R^{d_{\mathrm{model}}\times d_{k}}$, and $W^{V}\in R^{d_{\mathrm{model}}\times d_{v}}$ are the linearity transformation matrices. In MSA mechanism, multiple self-attention operations are run in parallel, and their concatenate outputs are returned.

(7) $$\mathrm{MSA}\left(S\right)=\mathrm{concat}(SA_{1}\left(S\right),\;\cdots ,\;SA_{h}(S))W^{O}$$

where the coefficient matrix $W^{O}\in R^{h\times d_{v}\times d_{\mathrm{model}}}$. In this research, $d_{k}=d_{v}=d_{\mathrm{model}}/h=4$, and we utilize h=3 concurrent attention heads. The computation process of the aforementioned multi-head self-attention mechanism is depicted in Figure 4.

The Transformer encoder used in this work consists of *L* layers, and the output of each layer serves as the input to the next layer. This process can be expressed by the following two equations:

(8) $$y_{i}=\mathrm{MSA}(\mathrm{LN}(S_{i-1}))$$

(9) $$S_{i}={\;S}_{i-1}+y_{i}+\mathrm{MLP}(\mathrm{LN}(y_{i}))$$

where i=1, ⋯, L and L=6 is chosen.

Finally, the max-pooling operation is performed along the first dimension of *S* to obtain the input of the MLP module, and pass the result through a softmax layer to output the probabilities for seizure and non-seizure.

### 2.3. Post-Processing

**Figure 5:**
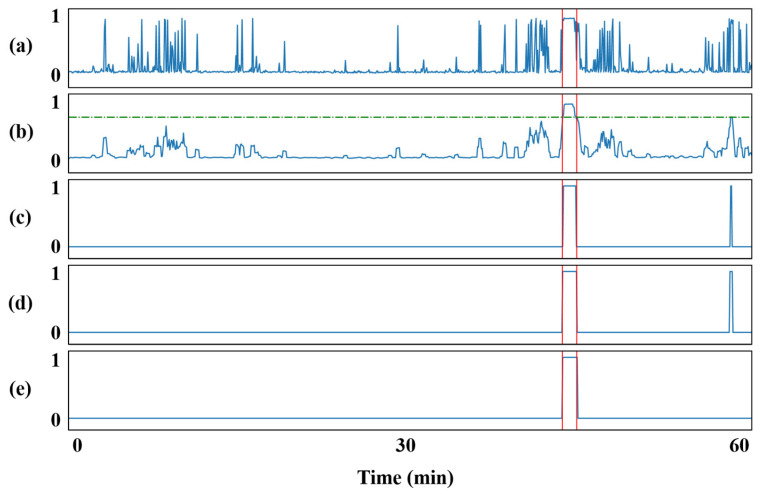
The post-processing procedure of 1-h EEG data. (**a**) The model prediction. (**b**) The outputs processed by MAF. (**c**) The binary values obtained from threshold determination. (**d**) The results after collar technology. (**e**) The final decisions after K-of-N discrimination. The vertical red lines represent expert-annotated seizure events, while the horizontal green line represents the threshold set during the binarization operation.

When using long-term continuous EEG recordings for event-based assessment, isolated false detections are often encountered. We apply moving average filtering (MAF), collar technique, and K-of-N method to reduce the false detection rate (FDR) of the algorithm. MAF is applied before thresholding to smooth the predicted scores. The collar technique is mainly used to prevent seizure segments that are correctly detected from being filtered out. In this study, K and N are set to 5 and 10, respectively, and a 40 s window consisting of N epochs is used to slide over the model prediction results. If five or more samples in the window are judged as seizures, the time span is considered a seizure period. The post-processing procedure is illustrated in Figure 5.

## 3. Experiments and Results

### 3.1. EEG Dataset

The long-term scalp EEG dataset used in this study was collected at the Children’s Hospital Boston, and consists of EEG recordings from pediatric subjects with intractable seizures [45,46]. The recordings, grouped into 24 cases, were collected from 23 subjects (5 males, ages 3–22, and 17 females, ages 1.5–19) [47]. All signals were sampled at 256 Hz with 16-bit resolution. Most files contain 23 EEG channels (24 or 26 in a few cases). The international 10–20 system of EEG electrode positions and nomenclature was used for these recordings. In summary, these records include 182 seizures (166 in the original set of 24 cases).

The details of the used CHB-MIT dataset are listed in Table 1. In this work, we evaluated the proposed model based on the segments for all patients. However, due to the fact that the duration of each seizure in chb16 is less than 15 s, this patient was excluded from the event-based evaluation of the model.

### 3.2. Experimental Process and Evaluation

In the segment-based experiments, each patient’s ictal EEG recordings were divided in 4 s seizure segments with a sliding window based on the start and end times of the seizures annotated by the experts, and an equal number of 4 s normal EEG segments were randomly selected. Since seizure EEG data is much less than normal EEG data and in order to enhance the system’s generalization ability, we used 50% overlapping sliding window when dividing the ictal data, while no overlap strategy was performed when dividing normal EEG data. After segmenting the dataset, we generated random seeds to shuffle the segmented dataset during model training. The dataset was then grouped into training and testing subsets with a 3:1 ratio.

For the segment-based evaluation, five metrics were introduced to assess the performance of the model: accuracy, sensitivity, specificity, precision, and area under the curve (AUC).

(10) $$\mathrm{Accuracy}=\frac{\mathrm{TN}+\mathrm{TP}}{\mathrm{TN}+\mathrm{FP}+\mathrm{FN}+\mathrm{TP}}$$

(11) $$\mathrm{Sensitivity}=\frac{\mathrm{TP}}{\mathrm{TP}+\mathrm{FN}}$$

(12) $$\mathrm{Specificity}=\frac{\mathrm{TN}}{\mathrm{TN}+\mathrm{FP}}$$

(13) $$\mathrm{Precision}=\frac{\mathrm{TP}}{\mathrm{FP}+\mathrm{TP}}$$

where, *TP* (true positive) and *TN* (true negative) refer to the number of ictal and non-ictal segments that are correctly recognized by our detection method, respectively. *FP* (false positive) denotes the number of non-ictal EEG segments incorrectly judged as ictal by the detection method, and *FN* (false negative) means that the ictal segment is labeled to be the non-ictal segment.

AUC stands for the area under the curve of the receiver operating characteristic curve (ROC), which denotes the probability that the positive samples are assigned a higher score than the negative samples [48].

In the event-based experiments, partial seizure events of each patient were used as training samples. The processing of the training set followed a similar approach to the segment-based processing, with the exception that the optimal model parameters were saved at the end. Thereafter, all recordings of the patient except for those training seizure events are used for testing. All the test files are arranged in chronological order according to the recorded time, and the onset as well as offset times of epileptic seizures are annotated based on the instruction files. After post-processing operations, the previously saved model’s output is considered a correct detection if it predicts a seizure within the onset and offset time range. Conversely, if the model predicts a seizure outside the range, it is considered a false alarm. CHB-MIT dataset A total of 865.15 h of EEG data containing 97 seizures from the CHB-MIT dataset were utilized for event-based performance testing, alone with other 64 seizures used for model training.

For the event-based evaluation, three measures are utilized to evaluate the model in clinical practice: sensitivity, FDR, and detection delay. Sensitivity is computed by dividing the number of correctly detections by the number of testing seizures for per patient. FDR represents the number of falsely detected seizures by the model within one hour. Detection delay is the time interval between the point at which the model makes a correct seizure detection and the expert-annotated onset time of the seizure.

### 3.3. Results

The model was implemented using PyTorch 1.13.1 in Python 3.9. All the results presented below were obtained through experiments conducted on a GPU configured with GeForce RTX 3050 from United States-based company NVIDIA.

Table 2 lists the segment-based experimental results. On average, the accuracy of 96.15%, the sensitivity of 96.11%, the specificity of 96.38%, the precision of 96.33%, and the AUC of 0.98 are achieved. There are more than half of the patients having an AUC above 0.99, while 11 patients have a sensitivity greater than 97%. Patients 15 and 21 have relatively low classification accuracies, both falling below 90%.

The event-based experimental results are shown in Table 3. For event-based evaluation, we obtained an average sensitivity of 96.57%, an average FDR of 0.38/h, and an average detection delay of 20.62 s. In addition, apart from patients 12, 15, 23, and 24, no seizure events were missed in the other patients. As for patient 16, the average duration of her epileptic seizures was only 8.4 s. Even with 50% overlapping sampling of 4 s segments, the training data were severely insufficient. Therefore, this patient was excluded from the event-based experiments in this study.

## 4. Discussion

### 4.1. Comparison with Existing Methods

Table 4 lists some state of the art seizure detection methods that have also been evaluated on the CHB-MIT EEG database. Ansari et al. [49] achieved a sensitivity of 85%, specificity of 89.06%, and classification accuracy of 89.06% on the CHB-MIT dataset by combining frequency domain features of EEG signals generated by wavelet packet decomposition with a neutrosophic logic-based k-means nearest neighbor (NL-k-NN) classifier. Janjarasjitt [50] extracted wavelet features from scalp EEG recordings and classified them using support vector machines (SVM), achieving an accuracy of 96.87%, sensitivity of 72.99%, and specificity of 98.13%. He et al. [51] used graph attention networks (GAT) and BiLSTM as the front-end for extracting spatial features and the back-end for exploring temporal relationships. Through extensive experiments, they demonstrated that this model can effectively detect epileptic seizures from raw EEG signals. The automatic seizure detection system proposed by Yao et al. [52], based on transfer learning of VGGNet-16 and gated recurrent unit (GRU), achieved sensitivity, specificity, and accuracy of 90.12%, 96.32%, and 96.31%, respectively. However, their experiments were conducted only on 12 patients. Cura et al. [53] computed features like higher-order joint time-frequency (HOJ-TF) moments and gray-level co-occurrence matrix (GLCM) through synchrosqueezing transform (SST) to obtain high-resolution time-frequency representations of EEG signals. These representations were combined with machine learning algorithms achieved a promising classification performance. Hu et al. [54] introduced local mean decomposition (LMD) and feature extraction processes to reduce computational complexity while maintaining the non-stationarity of EEG signals. They used BiLSTM to achieve a sensitivity of 93.61% and specificity of 91.85%. Duan et al. [55] proposed an epileptic seizure detection method based on deep metric learning, with an average accuracy of 86.68% and average specificity of 93.71% on the CHB-MIT dataset. Shyu et al. [56] presented an end-to-end deep learning model comprising an inception module and a residual module for seizure detection. Although their method achieved higher accuracy of 98.34% and specificity of 98.79% on the CHB-MIT database, it exhibited much lower sensitivity of 73.08%. Jiang et al. [57] used a seizure detection method based on the brain functional network structure and time-frequency multi-domain features. They employed SVM classifier for ictal EEG classification. However, their method operated on multi-domain hand-crafted features and irrelevant features needs to be eliminated using principal component analysis (PCA), which increase the complexity of the algorithm. Considering that seizure episodes have much shorter durations compared to non-seizure EEG, Gao et al. [58] utilized generative adversarial network (GAN) for data augmentation, and used one-dimensional convolutional neural network (1DCNN) for seizure detection, achieving a sensitivity of 93.53% and specificity of 99.05%. Their achieved overall sensitivity is lower than our method.

Most of the aforementioned studies only employed segmented EEG for evaluation. Event-based assessments for epileptic seizure detection are more concordant with practical clinical applications and proved to be challenging due to the frequent appearance of artifacts in long-term continuous EEG. Zhang et al. [59] combined wavelet transform and bidirectional gated recurrent unit (Bi-GRU) network followed by certain post-processing steps, achieving an average sensitivity of 93.89% and an average specificity of 98.49%. Among the 128 seizure events used, the model only missed four detections and reduced the false alarm rate to 0.31 per hour, indicating the potential superiority of the Bi-GRU network in long-term EEG applications. Yoshiba et al. [60] yielded a detection delay of 7.39 s by using a single EEG channel combined with pretrained ResNet. However, this study only used data from 10 patients (3–19 years old). Samiee et al. [61] proposed a feature extraction method based on sparse rational decomposition and Local Gabor Binary Patterns (LGBP), with a sensitivity of 91.13% and FDR of 0.35/h at event-based level and a delay of 5.98 s. Compared with the above research, our proposed method obtained the highest event-based sensitivity within a competitive FDR.

In previous studies, CNNs have been employed for encoding and classifying EEG features. For instance, Sun et al. [62] proposed a subject transfer neural network (STNN) by integrating CNN with self-attention, achieving satisfactory results in motor imagery classification tasks. However, the local convolutional structure in CNNs poses difficulty to capture the global features of input signals. Meanwhile, CNNs require serial operations at each time step, resulting in lower computational efficiency when handling long-term time-series data. In contrast, the Transformer encoder with multi-head self-attention utilized in this work can capture long-range correlations and global features of input signals, and allows for parallel computation and better classification capability. Moreover, in comparison with the continuous wavelet transform (CWT) used by Sun et al. [62], the S-transform adopted in this method combines the advantages of CWT and Short-Time Fourier Transform (STFT) while maintaining lower computational complexity, enabling better extraction of time-frequency features from EEG signals.

Overall, the performance and stability of the proposed method are satisfactory. These results verify the effectiveness of the combination of S-transform and Transformer in epileptic seizure detection.

### 4.2. Visualization of t-Distributed Stochastic Neighbor Embedding (t-SNE)

**Figure 6:**
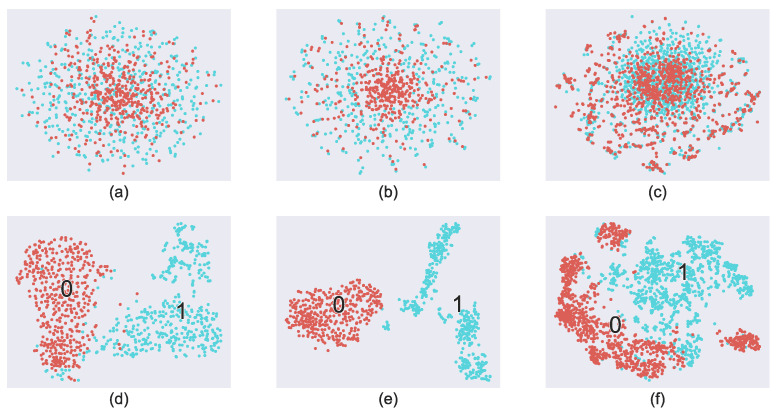
*t*-SNE visualization of samples from chb08, chb11, and chb15. (**a**–**c**) Distributions of original EEG samples from chb08, chb11, and chb15. (**d**–**f**) Corresponding distributions of those EEG samples after S-transform. The red points labeled with the number 0 represent normal samples while the blue points labeled with the number 1 represent epileptic samples.

Figure 6 illustrates the visualization of the sample distribution obtained from seizure and non-seizure samples extracted from the EEG recordings of three patients (chb08, chb11, chb15) using the *t*-SNE algorithm. The upper three plots (a), (b), and (c) depict the two-dimensional projection of the 4 s original EEG segments from the aforementioned three patients, while the bottom three plots (d), (e), and (f) show the corresponding sample distributions in the time-frequency domain after applying the S-transform. In these plots, the red points represent normal samples while the blue points represent epileptic samples. It is evident from Figure 6c,f that the EEG time-frequency features obtained by the S-transform exhibit better separability when compared to the scattered distribution of the original EEG samples embedded in time domain. These observations indicate the effectiveness of the S-transform in assisting to feature extraction from normal and epileptic EEGs. However, these time-frequency features are not completely separable, necessitating further extraction and classification with the assistance of the Transformer Encoder. To achieve better seizure detection performance, we incorporated S-transform and Transformer in this work.

### 4.3. Attention to EEG Channels

**Figure 7:**
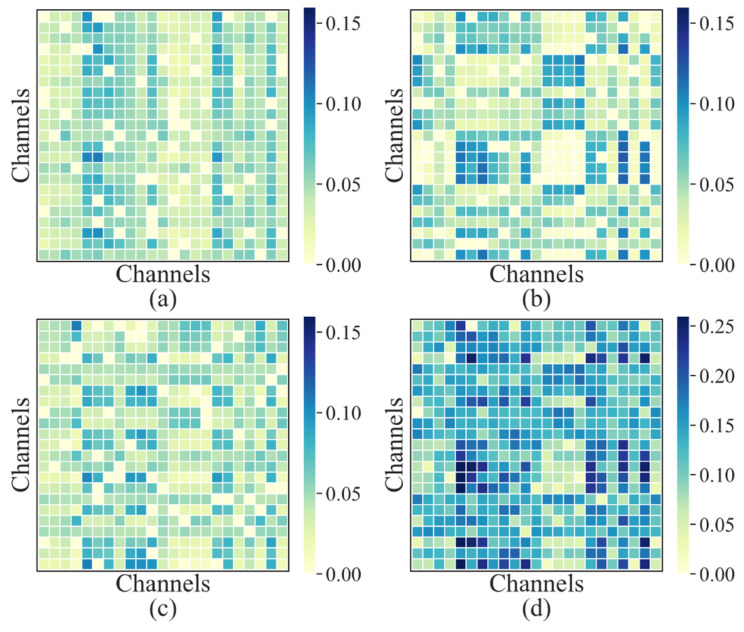
The visualization of attention mechanism. (**a**–**c**) The channel weight matrix generated by the multi-head attention of the last encoder layer of Transformer. (**d**) The sum of the above three.

**Figure 8:**
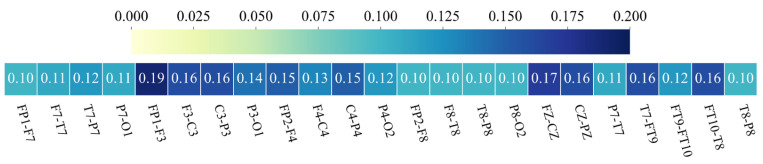
The attention weights assigned to each EEG channel for patient 23.

In recent years, some studies have aimed not only to achieve high classification accuracy and sensitivity but also to determine which channels of multi-channel EEG recordings related to seizure onsets [63]. In the proposed Transformer encoder, the self-attention module can characterize the active channels by quantitating EEG channel attention weights. Figure 7 illustrates the attention weight matrices generated by the last encoder layer of the proposed model for each of the three attention heads on patient chb23, along with their sum. The final output of the Transformer encoder is the product of the weight matrix (*W*) and the input (*S*). By summing and averaging the elements in each column vector in *W,* the channel attention weight vector can be obtained. As shown in Figure 8, the model assigns higher attention to channels FP1-F3, F3-C3, C3-P3, FZ-CZ, CZ-PZ, T7-FT9, and FT10-T8, indicating that the epileptic EEG activity is likely to be active in these channels.

### 4.4. Future Work

Due to the significant individual variability observed in the severity and duration of epileptic seizures, extensive research of epileptic detection, including the present study, has focused on individualized patient-specific methods. However, a critical avenue for future research lies in enhancing the generalization and capabilities of the model to make it more suitable for real-world clinical application. To this end, exploration into cross-subject epileptic detection is in critical need. Our future endeavors will involve conducting more experiments to further verify the generalization performance of the proposed model for patient-independent seizure detection, extending its applicability even in across-dataset scenarios.

## 5. Conclusions

In this study, we propose a novel automatic seizure detection approach based on S-transform and Transformer. The S-transform enables more comprehensive time-frequency representations compared to STFT and wavelet transform, facilitating the Transformer encoder to learn more distinctive features. Meanwhile, the proposed Transformer model can assign unequal attention weights to different EEG channels, thereby extracting spatial features of multi-channel EEG signals and enhancing the interpretability of the model by preserving the original labels of the channels. The method has been investigated on the CHB-MIT database and achieves 96.15% accuracy, 96.11% sensitivity, 96.38% specificity, 96.33% precision, and 0.98 AUC on segment-based evaluation. Additionally, sensitivity of 96.57%, 0.38/h FDR, and 20.62 s average latency are yielded under event-based level. These outstanding results indicate the feasibility of implementing this seizure detection method in clinical applications.

## Figures and Tables

**Table 1:** Details of the used CHB-MIT EEG dataset.

Patient	Gender	Age (Years)	No. of Used Channels	No. of Epileptic Events	Duration of Epileptic Seizures (s)
1	F	11	23	7	442
2	M	11	23	3	172
3	F	14	23	7	402
4	M	22	23	4	378
5	F	7	23	5	558
6	F	1.5	23	10	138
7	F	14.5	23	3	325
8	M	3.5	23	5	919
9	F	10	23	4	276
10	M	3	23	7	447
11	F	12	23	3	806
12	F	2	23	40	1475
13	F	3	18	12	535
14	F	9	23	8	109
15	M	16	18	20	1992
16	F	7	18	10	84
17	F	12	23	3	293
18	F	18	23	6	317
19	F	19	23	3	236
20	F	6	23	8	294
21	F	13	23	4	199
22	F	9	23	3	204
23	F	6	23	7	424
24	-	-	23	16	511

**Table 2:** Detection results of the proposed method on segment-based metrics.

Patient	Accuracy (%)	Sensitivity (%)	Specificity (%)	Precision (%)	AUC
1	99.11	98.21	100	100	1
2	97.73	95.24	100	100	0.9917
3	95.05	96	94.12	94.12	0.9694
4	96.88	97.56	96.36	95.24	0.9849
5	99.29	98.73	100	100	1
6	97.22	94.44	100	100	0.9938
7	96.3	95.24	97.44	97.56	0.9939
8	97.40	97.30	97.50	97.30	0.9877
9	100	100	100	100	1
10	98.25	98.39	98.08	98.39	0.9932
11	96.53	97.73	95.61	94.51	0.9929
12	97.10	96.00	98.28	98.36	0.9919
13	96.92	95.45	98.44	98.44	0.9744
14	97.78	100	96.30	94.74	0.9979
15	89.02	84.21	93.88	93.27	0.9377
16	100	100	100	100	1
17	93.06	94.59	91.43	92.11	0.9521
18	93.67	90.48	97.30	97.44	0.9665
19	96.61	100	93.75	93.10	0.9954
20	94.44	92.68	96.77	97.44	0.9929
21	83.67	95.65	73.08	75.86	0.8094
22	96.08	95.65	96.43	95.65	0.9984
23	97.14	94.55	100	100	0.9975
24	98.39	98.44	98.33	98.44	0.9982
Total	96.15	96.11	96.38	96.33	0.98

**Table 3:** Detection results of the proposed method on event-based metrics.

Patient	Test Set Duration (h)	No. of Training Seizures	No. of Testing Seizures	No. of True Detections	Sensitivity (%)	FDR (%)	Latency (s)
1	37.38	3	4	4	100	0.11	19
2	34	2	1	1	100	0	36
3	34	4	3	3	100	0.56	16.3
4	149.38	2	2	2	100	0.39	9.5
5	37	2	3	3	100	0	33.3
6	48.52	4	3	3	100	1.26	6
7	63.05	1	2	2	100	0.03	19.5
8	19	1	4	4	100	0.58	64.25
9	62.27	2	2	2	100	0.61	20.5
10	44	3	4	4	100	0	8.75
11	33.79	1	2	2	100	0.06	4
12	16.67	4	15	12	80	0.42	3.08
13	29	4	7	7	100	0.86	19.14
14	22	4	4	4	100	0.05	9.5
15	34	6	14	11	78.57	0.71	21.09
17	19	2	2	2	100	0.05	41.5
18	31.63	4	3	3	100	0.41	15
19	27.93	2	2	2	100	0.11	11
20	24.63	3	4	4	100	0.77	6.75
21	31	2	2	2	100	0.97	37.25
22	30	1	2	2	100	0.17	23
23	21.6	1	4	3	75	0.69	31.33
24	15.3	6	8	7	87.5	0	18.43
Total	865.15	64	97	89	96.57	0.38	20.62

**Table 4:** Performance comparison of different seizure detection methods reported for CHB-MIT dataset.

Author	Method	Segment-Based					Event-Based		
		Accuracy (%)	Sensitivity (%)	Specificity (%)	Precision (%)	AUC (%)	Sensitivity (%)	FDR (/h)	Latency (s)
Ansari et al. [49]	kNN	89.06	85	89.06	-	-	-	-	-
Janjarasjitt et al. [50]	Wavelet + SVM	96.87	72.99	98.13	-	-	-	-	-
He et al. [51]	GAT + BiLSTM	98.52	97.75	94.34	-	96.81	-	-	-
Yao et al. [52]	Transfer learning + GRU	96.31	90.12	96.32	-	-	-	-	-
Cura et al. [53]	SST + kNN	95.1	90.3	-	93.4	-	-	-	-
Hu et al. [54]	LMD + BiLSTM	-	93.61	91.85	-	-	-	-	-
Duan et al. [55]	Deep metric learning	86.68	79.64	93.71	-	-	-	-	-
Shyu et al. [56]	Inception and Residual model	98.34	73.08	98.79	-	-	-	-	-
Jiang et al. [57]	PMNet + SVM	96.67	97.72	95.62	-	-	-	-	-
Gao et al. [58]	GAN + 1DCNN	93.53	99.05	-	-	-	-	-	-
Zhang et al. [59]	Bi-GRU	98.49	93.89	98.49	-	-	95.49	0.31	-
Yoshiba et al. [60]	ResNet	-	88.73	98.98	-	-	-	-	7.39
Samiee et al. [61]	Sparse rational decomposition + LGBP	-	70.40	99.10	-	-	91.13	0.35	5.98
This work	S-transform + Transformer	96.15	96.11	96.38	96.33	98	96.57	0.38	20.62

## Data Availability

The CHB-MIT Database analyzed in this study is available from https://physionet.org/content/chbmit/1.0.0/.
